# Biological activity of galacto-oligosaccharides: A review

**DOI:** 10.3389/fmicb.2022.993052

**Published:** 2022-09-06

**Authors:** Zhaojun Mei, Jiaqin Yuan, Dandan Li

**Affiliations:** ^1^Department of Pediatrics, Luzhou Maternal and Child Health Hospital, Luzhou Second People’s Hospital, Luzhou, China; ^2^Department of Orthopedics, The Second People’s Hospital of Yibin, Yibin, China; ^3^University of Chinese Academy of Sciences, Beijing, China

**Keywords:** galacto-oligosaccharides, prebiotics, β-galactosidase, biological activity, ulcerative colitis

## Abstract

Galacto-oligosaccharides (GOS) are oligosaccharides formed by β-galactosidase transgalactosylation. GOS is an indigestible food component that can pass through the upper gastrointestinal tract relatively intact and ferment in the colon to produce short-chain fatty acids (SCFAs) that further regulate the body’s intestinal flora. GOS and other prebiotics are increasingly recognized as useful food tools for regulating the balance of colonic microbiota-human health. GOS performed well compared to other oligosaccharides in regulating gut microbiota, body immunity, and food function. This review summarizes the sources, classification, preparation methods, and biological activities of GOS, focusing on the introduction and summary of the effects of GOS on ulcerative colitis (UC), to gain a comprehensive understanding of the application of GOS.

## Overview of functional oligosaccharides

Oligosaccharides are a class of oligomeric saccharides composed of 2–10 identical or different monosaccharides linked by glycosidic bonds to form straight or branched chains (Dai et al., 2018). According to whether it can be degraded by gastric acid and whether it has special physiological functions to the human body, oligosaccharides can be divided into ordinary oligosaccharides and functional oligosaccharides (Hsu et al., 2007; Wilson and Whelan, 2017). Ordinary oligosaccharides include lactose, sucrose and maltose, which can be digested and absorbed by the body and mainly provide the body with the energy needed to maintain life activities. Functional oligosaccharides mainly refer to GOS, fructo-oligosaccharides, xylo-oligosaccharides, isomaltose oligosaccharides, etc., which cannot be digested and absorbed by the body, but directly enter the intestinal tract to be utilized by *Bifidobacterial* (Macfarlane et al., 2008; Vera et al., 2016). Compared with ordinary oligosaccharides, functional oligosaccharides have the following unique physiological functions: preventing diarrhea or intestinal obstruction, regulating intestinal flora, promoting the proliferation of *Bifidobacteria*, preventing cancer, positive effect on lipid metabolism, stimulating mineral absorption, and immunomodulatory properties. The concept of prebiotics was first proposed by Gibson and Roberfroid (1995), and the so-called prebiotics mainly refer to functional oligosaccharides (Gibson and Roberfroid, 1995; Sangwan et al., 2011). Galacto-oligosaccharides (GOS) is a functional oligosaccharide with natural properties. A mixture of GOS and fructo-oligosaccharides was introduced into the market, especially for infant formula, stimulates the development of the same intestinal microorganisms as breastfed infants, showing a pronounced prebiotic effect (Boon et al., 2000). Therefore, biocatalytically generated GOS is of interest for infant nutrition.

## Galacto-oligosaccharides

### Classification of galacto-oligosaccharides

GOS is an oligosaccharide with natural functions, an important active substance in milk, and a commonly used prebiotic (Tian et al., 2019; Mistry et al., 2020). GOS is divided into α-galacto-oligosaccharides (α-GOS) and β-galacto-oligosaccharides (β-GOS) due to the different galactosidic bonds attached (Tian et al., 2019). To date, the most used for research and commercial production is β-GOS, which is synthesized by β-galactosidase through the lactose transgalactosylation reaction. However, there are currently few studies on α-GOS. α-GOS is an oligosaccharide widely distributed in various plants (Dai et al., 2018).

### Preparation of galacto-oligosaccharides

At present, the production methods of GOS mainly include the following (Weijers et al., 2008; Torres et al., 2010): (1) Natural extraction, GOS is mainly produced by the hydrolysis of lactose or whey. GOS has few sources in nature, mainly extracted from the seeds of leguminous plants, and does not contain charges, so it is difficult to separate and extract GOS from natural components; (2) Hydrolyzed polysaccharides, polysaccharide hydrolysis has the disadvantages of low conversion rate and complex components, and it is not easy to obtain GOS products with high content; (3) Chemical synthesis, the required reagents are toxic and easy to remain, and the synthesis process may cause pollution, so it is not suitable for food grades production of GOS; (4) Enzymatic synthesis, GOS is mainly synthesized through the transglycosidase of β-galactosidase. The enzymatic reaction has the advantages of safety and high efficiency, and does not cause environmental pollution, and is currently the main method for the production of GOS. β-galactosidase can be derived from various microorganisms (Weijers et al., 2008). The synthesis of GOS is usually highly influenced by several factors, including enzyme source, lactose concentration, substrate composition, and reaction conditions, etc. (Fewtrell et al., 2007). There is no doubt that β-galactosidase will develop into a promising synthetic tool.

### Structure and physicochemical properties of galacto-oligosaccharides

GOS are galactose-containing oligosaccharides whose chemical structures vary by chain length, branching, and glycosyl linkages. Depending on the source of β-galactosidase, using lactose as a single substrate, the galactose released during the enzymatic hydrolysis of lactose can be transferred to another lactose molecule through β(1→6), β(1→4) or β(1→3) glycosidic linkage to the galactose moiety (Varney et al., 2017; Lu et al., 2020). For example, β-galactosidases from *Kluyveromyces lactis* and *Aspergillus oryzae* mainly produce β-1,6-linked GOS (Figueroa-González et al., 2011). In contrast, β-galactosidase from *Bacillus circulans* mainly produces β-1,4-linked GOS (Otieno, 2010). In general, GOS is a colorless water-soluble product with a viscosity similar to corn syrup (Kimura et al., 2015). GOS is stable at pH 7, and thermal stability is due to the presence of β-glucosidic bonds between the moieties. The sweetness of GOS is generally lower, usually 0.3–0.6 times that of sucrose (Panesar et al., 2018). Therefore, GOS can be used as a filler in various foods to enhance the flavor of other foods. Because GOS is not easily decomposed in the upper gastrointestinal tract and has a low caloric value, it is suitable for low-calorie diets and diabetic patients. Besides, GOS has a high moisturizing ability (Li et al., 2009).

### Biological activity of galacto-oligosaccharides

GOS enhances health-related short-chain fatty acids (SCFAs) production, growth and differentiation of colonic epithelial cells, energy transduction in colonocytes, lipid and carbohydrate metabolism, reduces the number of potentially pathogenic bacteria, and promotes intestinal normal function, etc. (Farthing, 2004). A research shows (He et al., 2021a) that the addition of GOS to a high-fat diet can effectively stimulate the growth of *Bifidobacteria*. Most *Bifidobacteria* can produce lactic acid, which can acidify the intestinal environment, limit the growth of pathogenic bacteria, and improve the mucosal barrier function (Weijers et al., 2008).

#### Galacto-oligosaccharides and intestinal flora

The human colon contains approximately 1,014 different bacterial species, and the gut microbiota consists of approximately 500 different anaerobic species (Sheveleva, 1999). The metabolic activities of these bacteria may have dramatic effects on the physiological processes of the human host (Eiwegger et al., 2004). Increased numbers of *Bifidobacteria* in the gut can antagonize the activity of spoilage bacteria such as *Clostridium* spp., thereby reducing the formation of toxic fermentation products (Macfarlane et al., 2008). In healthy individuals, there is a balance between gut commensal microbiota and pathogenic bacteria. When this balance is disrupted, intestinal barrier function is impaired (Farthing, 2004). The breakdown of gut barrier function and immune function is associated with the development of multiple organ dysfunction syndrome and systemic inflammatory response syndrome (Eiwegger et al., 2004). In this case, the symbiotic relationship between the host and the microbiota can be improved by prebiotics in the gut. Ulcerative colitis (UC) is a chronic inflammatory disease that occurs in the colonic mucosa, and the pathogenesis may be related to intestinal flora, immunity, and genetics (Ordás et al., 2012). UC patients are prone to repeated diarrhea, pus and blood in the stool, abdominal pain, tenesmus, and even increase the risk of colorectal cancer (Jess et al., 2012). Currently, the treatment of UC includes 5-aminosalicylic acid drugs, steroids and immunosuppressants (Chen et al., 2020), but there are serious side effects (Roselli et al., 2022). GOS has received increasing attention due to its better biological activity. As shown in Table 1, the research situation of GOS on UC in recent years was summarized.

**TABLE 1:** Summarize the experimental and clinical research results of GOS in the treatment of UC.

GOS sources	Induced colitis	Intervention	Results	References
Commercialized GOS	Mice with 5% DSS^a^	GOS (0.5 g/kg/d) group, GOS (0.5 g/kg/d) + DSS group	GOS attenuated DSS-induced weight loss, colon histological damage, and reduces the secretion of cytokines in colon tissue.	Chu et al., 2020
Commercialized GOS	Mice with DSS	GOS (400 and 800 mg/kg) + DSS group	GOS can improve DSS-induced colitis symptoms, such as weight loss, reduced colon shortening.	Park et al., 2021
Enzymatic-synthesized GOS	Mice with 2% DSS	α-GOS (400 mg/kg/day) + DSS group and β-GOS (400 mg/kg/day) + DSS group	α-GOS and β-GOS could inhibit the activation of the NOD-like receptor (NLR) family member NLRP3 inflammasome-mediated inflammation.	He et al., 2021b
Commercialized GOS	Smad3-deficient mice	Model group and Smad3^–/–^ mouse were given GOS (5,000 mg/kg body weight)	GOS enhances growth of *Bifidobacterium*, alters NK cells in the spleen and MsLN, stimulates IL-15 production in proximal colon tissue.	Gopalakrishnan et al., 2012
Commercialized GOS	Rats with TNBS	GOS-1 or GOS-2 (4 g/kg/day)	GOS increased levels of *Bifidobacteria* and other bacteria in the colon, but did not reduce the severity of inflammation.	Holma et al., 2002
Commercialized GOS	SD rats (Rag2^–/–^) with *helicobacter hepaticus*	GOS (5,000 mg/day/kg) group, GOS + miR-19b inhibitor groups	GOS can prevent diarrhea and inflammation caused by Rag2 -/- rat anti-*Helicobacter* injection and inhibit the release of pro-inflammatory cytokines.	Sun et al., 2019
Vegetal compounds (LA-GOS)	Mice with 2% DSS	LA-GOS (2.5 g/kg bw/day), and the treated DSS group (UC + LA-GOS)	LA-GOS alleviated histopathological damage, decreased intestinal pH, and increased the abundance of beneficial bacteria in the UC group.	Godínez-Méndez et al., 2021
Synbiotics	Patients with UC	Group was treated with the synbiotics	Synbiotics improved clinical symptoms in UC patients and decreased fecal number and fecal pH in *Bacteroidetes*.	Ishikawa et al., 2011
Commercialized GOS	Patients with UC	UC patients with the GOS (2.8 g/d)	Prebiotics did not lower clinical scores or inflammation but normalized stools.	Wilson et al., 2021

^a^Dextran Sulfate Sodium.

One of the mechanisms by which GOS exerts its antibacterial effect is that it can adhere to the binding sites of bacteria on the surface of enterocytes, thereby preventing the adhesion of harmful microorganisms (Dwivedi et al., 2016). GOS has been shown to increase the number of *Bifidobacterium* and *Bacteroides* in rats by qPCR technique (Marín-Manzano et al., 2013). Breastfeeding is the natural way to get the best gut flora. Results obtained in formula-fed infants showed that GOS was able to induce the development of gut microbiota similar to breast-fed infants (Boehm et al., 2002). SCFAs are the end products of fermentation by microorganisms such as *Bifidobacterium* and *Lactobacillus*. In an *in vitro* test, fecal microbiota obtained from breastfed infants produced the same SCFAs as those obtained from supplemental GOS feeding (Knol et al., 2005). A clinical study (Moro et al., 2002) showed that supplementation of GOS at a ratio of 90–10% produced bifidobacterial effects similar to breastfeeding. In animal feeding experiments, the addition of prebiotic GOS to standard enteral nutrition in rats with severe acute pancreatitis improved intestinal barrier function (Zhong et al., 2009). The addition of GOS to a commercial basal diet demonstrated an increase in the density of *Bifidobacterial* (Walton et al., 2012). A recent study investigated the effect of GOS on the microbiota composition and immune function of healthy elderly volunteers, and GOS administration resulted in a significant reduction in the number of harmful bacteria and a significant increase in the number of beneficial bacteria, especially *Bifidobacterial* (Vulevic et al., 2008). In conclusion, there is a growing body of research demonstrating that GOS modulates the gut microbiota and stimulates the immune system in the same way as breastfeeding.

#### Effects of galacto-oligosaccharides on skin

Ingestion of prebiotics is not only beneficial for the gut, but also for the improvement of allergic skin (Hong et al., 2015). A questionnaire survey found that women with abnormal bowel movements had many skin problems, such as dry skin (Kawakami et al., 2005). Studies have shown that oral administration of *Lactobacillus* improves atopic dermatitis in clinical trials and protects the immune homeostasis of the skin after UV exposure (Peguet-Navarro et al., 2008). Phenols produced by gut bacteria were found to accumulate in the skin through circulation and disrupt keratinocyte differentiation in hairless mice (Kalliomäki et al., 2003). GOS promises to restore skin health by reducing the production of phenols by the gut microbiota. In women, consumption of GOS eliminated the loss of water and keratin caused by phenolics (Kano et al., 2013). Consumption of prebiotics reduces the severity of allergic skin diseases. A randomized controlled study showed that a mixture of GOS in healthy infants prevented the development of atopic dermatitis (Moro et al., 2006). GOS can block atopic dermatitis in mice by inducing the production of IL-10 and inhibiting the production of IL-17 (Tanabe and Hochi, 2010). van Hoffen et al. (2009) showed that GOS supplementation in high-risk infants reduced total immunoglobulin responses, modulated allergic responses, and reduced Ig E.

#### Galacto-oligosaccharides and calcium absorption

Several studies in animals and humans have shown that GOS has a positive effect on bone composition and structure (Weaver et al., 2011). Several mechanisms have been proposed (Griffin et al., 2002): (1) Bacterial fermentation of acidic metabolites in the colon reduces the local pH of the intestine, thereby increasing the luminal concentration of calcium ions and increasing passive calcium absorption; (2) SCFAs modify the charge of calcium, promote calcium channels, and increase calcium absorption. One study (Scholz-Ahrens et al., 2002) demonstrated in ovariectomized rats and pigs that administration of GOS decreased intestinal pH, increased bone mineralization, inhibited estrogen-deficiency-induced bone degradation, and preserved bone structure. The beneficial effects of GOS on calcium absorption and bone mineralization have also been demonstrated in postmenopausal women (Abrams et al., 2005).

#### Relieve lactose intolerance and prevent constipation

In addition to the protection of intestinal barrier function, GOS also plays an important role in alleviating lactose intolerance and preventing constipation. A clinical trial study demonstrated that women with constipation took GOS for 3 weeks to significantly relieve constipation symptoms (Teuri and Korpela, 1998). Prebiotics increase the water-binding capacity of the gut. These movements increase stool weight and frequency and also soften stools, resulting in reduced transit time. Different levels of GOS in infant formula increase stool frequency (Ben et al., 2008). Compared with infants receiving standard formula, infants receiving prebiotic supplements had softer stools and stool consistency similar to that of breastfed infants (Schmelzle et al., 2003; Fedorak and Madsen, 2004).

#### Reduce the risk of cancer

The effect of GOS and other prebiotics on tumors is not well understood. The gut microbiota plays an important role in dietary metabolism, and this bacterial metabolite affects colon cancer development and progression (Duerr and Hornef, 2012). A study by Studies have shown that intestinal prebiotic fermentation products can inhibit the growth of colon cancer cells (Shoaf et al., 2006; Fernández et al., 2018). The effects of prebiotics on intestinal immune responses are achieved by stimulating intestinal epithelial cells. GOS has been shown to have a positive effect on immune function. GOS can induce the proliferation of *Lactobacillus* and *Bifidobacterium*, and its metabolites, SCFAs, play an important role in anticancer immune mechanisms such as colonic epithelial cell metabolism, gene expression, and induction of apoptosis (Rossi et al., 2018; De Almeida et al., 2019). Fernández et al. (2018) fed 10% GOS to colon cancer model mice. After 20 weeks of analysis of the colon tissue of the mice, it was found that feeding GOS significantly reduced the number of colon tumors. Meanwhile, metagenomic sequencing showed that the number of pro-inflammatory bacteria was reduced and the number of beneficial bacteria was significantly increased. In a clinical trial, GOS supplemented older adults found an increase in the immunomodulatory cytokine IL-10 and a decrease in IL-1β compared with placebo (Vulevic et al., 2015).

#### Regulation of lipid metabolism

GOS has low sweetness, less calories, and is not easy to be converted into cholesterol and fat, so it can improve lipid metabolism, reduce serum total cholesterol, increase the proportion of high-density lipoprotein in serum, and then effectively prevent and treat hypertension and hyperlipidemia. One study found that serum total cholesterol, LDL, and triglycerides were significantly lower in GOS-fed rats compared to controls (Hashmi et al., 2016).

## Application of galacto-oligosaccharides

Prebiotic supplementation in infant formula achieves the same gut flora as breastfeeding. GOS occurs naturally in breast milk and is an ideal ingredient in infant formula. Supplementation of low levels of GOS to infant formula improves stool frequency, reduces stool pH, and stimulates intestinal *Bifidobacteria* and *Lactobacilli*, as in breastfed infants (Ben et al., 2008). One study found that feeding infant formula with a small amount of GOS (0.24 g/dL) for 3 months stimulated the growth of *Bifidobacteria* and *Lactobacilli*, but did not harm the growth of *E. coli*. In another study of 365 healthy term infants at 8 weeks of age, the effect of higher GOS concentrations (0.44 and 0.50 g/dL) on the infant gut microbiome was investigated. The results showed that the addition of GOS to infant milk powder increased the number of *Bifidobacteria* and reduce the number of harmful bacteria (Ben et al., 2004; Sierra et al., 2015). GOS can be easily added to dairy applications such as yogurt and dairy beverages due to its excellent solubility. After adding GOS, the structure of the yogurt became smoother and more delicate. Also, the bacteria in the yogurt will not be destroyed. Due to the stability of GOS, it can be easily incorporated into juices and beverages, and the taste of beverages will not be affected when GOS is added (Kukkonen et al., 2007). Since prebiotics can be utilized by intestinal *Lactobacilli* and *Bifidobacteria*, while harmful bacteria can hardly be utilized, probiotics have significantly better therapeutic effect on digestive system diseases than antibiotics, so they have become their preferred drugs. GOS is recognized as a natural ingredient with prebiotic properties, and numerous studies reveal its health-promoting and food-functional properties, making it a potential choice as a nutritional supplement or food ingredient. The popularity of prebiotics in the functional food market is rapidly rising. More recently, most prebiotic ingredients have been used in breakfast breads, baked goods, and snacks. The rise in obesity and other metabolic-related health problems is rapidly encouraging consumers to focus on low-carb, well-balanced healthy diets, which in turn is driving demand for prebiotics. GOS and other prebiotics have been recommended for various human foods such as baked goods, sweeteners, yogurt, etc.

## Overview of β-galactosidase

β-Galactosidase, also known as lactase, can hydrolyze lactose into glucose and galactose, relieve lactose intolerance, and can also generate GOS through transglycosylation (Park and Oh, 2010a). In recent years, the isolation and identification of novel β-galactosidases have attracted attention. β-galactosidase with transgalactosylation activity is widely distributed in various microorganisms, including bacteria, archaea, yeast and fungi (Kim et al., 2004). Among various sources, β-galactosidases from *Aspergillus* and *Kluyveromyces* have been extensively studied due to their high lactose hydrolytic activity (Xiao et al., 2019). Most β-galactosidase must be secreted into the extracellular medium, thus requiring chemical and/or mechanical treatment to disrupt cell wall extractive enzymes (Yang and Silva, 1995). The most common bacterial source of β-galactosidase is from *Escherichia* coli (encoded by the lacZ gene) (Pignatelli et al., 1998). The *LacZ* gene has been cloned into different recombinant yeast expression systems. Lactose intolerance is a common problem, with more than 70% of the world’s population estimated to have problems digesting lactose, so there is a sizeable market for lactose-free milk and dairy products. GOS is also increasingly used in functional foods, such as fermented dairy products, bread, and beverages (Gasteiger et al., 2003; Park and Oh, 2010b). Recombinant β-galactosidase is also widely used as a fusion protein in different fields due to its easy detection, and one of the most relevant applications is biosensors (Huang et al., 2009). There is no doubt that β-galactosidase will develop into a promising synthetic tool.

## Conclusion

GOS is recognized as a natural ingredient with prebiotic properties. Adding prebiotics to your diet has been shown to be a better way to keep your gut flora balanced. In fact, GOS is marketed as a mixture of galactosyl oligosaccharides with different degrees of polymerization and configurations. Most studies on prebiotic utilization have been conducted *in vitro*, but the growth effects in these experiments are not necessarily replicated in the gut. Human studies on the effects of prebiotics (GOS), especially on infant growth and nutritional development, are a new direction for future research. Due to the key role of GOS in the functional food field, screening for β-galactosidase with transgalactosylation will undoubtedly attract great attention of researchers.

## Author contributions

ZM and JY organized the literature. ZM and DL co-authored the article. All authors contributed to the article and approved the submitted version.

## References

[B1] Abrams S. A. Griffin I. J. Hawthorne K. M. Liang L. Gunn S. K. Darlington G. (2005). A combination of prebiotic short- and long-chain inulin-type fructans enhances calcium absorption and bone mineralization in young adolescents. *Am. J. Clin. Nutr.* 82 471–476. 10.1093/ajcn.82.2.471 16087995

[B2] Ben X. M. Li J. Feng Z. T. Shi S. Y. Lu Y. D. Chen R. (2008). Low level of galacto-oligosaccharide in infant formula stimulates growth of intestinal Bifidobacteria and Lactobacilli. *World J. Gastroenterol.* 14 6564–6568. 10.3748/wjg.14.6564 19030213 PMC2773347

[B3] Ben X. M. Zhou X. Y. Zhao W. H. Yu W. L. Pan W. Zhang W. L. (2004). Supplementation of milk formula with galacto-oligosaccharides improves intestinal micro-flora and fermentation in term infants. *Chin. Med. J.* 117 927–931. 15198901

[B4] Boehm G. Lidestri M. Casetta P. Jelinek J. Negretti F. Stahl B. (2002). Supplementation of a bovine milk formula with an oligosaccharide mixture increases counts of faecal bifidobacteria in preterm infants. *Arch Dis. Child Fetal. Neonatal. Ed* 86:F178–F181. 10.1136/fn.86.3.f178 11978748 PMC1721408

[B5] Boon M. A. Janssen A. E. van ’t Riet K. (2000). Effect of temperature and enzyme origin on the enzymatic synthesis of oligosaccharides. *Enzyme Microb. Technol.* 26 271–281. 10.1016/s0141-0229(99)00167-2 10689088

[B6] Chen M. Ding Y. Tong Z. (2020). Efficacy and Safety of Sophora flavescens (Kushen) Based Traditional Chinese Medicine in the Treatment of Ulcerative Colitis: Clinical Evidence and Potential Mechanisms. *Front. Pharmacol.* 11:603476. 10.3389/fphar.2020.603476 33362558 PMC7758483

[B7] Chu H. Tao X. Sun Z. Hao W. Wei X. (2020). Galactooligosaccharides protects against DSS-induced murine colitis through regulating intestinal flora and inhibiting NF-κB pathway. *Life Sci.* 242:117220. 10.1016/j.lfs.2019.117220 31881230

[B8] Dai Z. Lyu W. Xiang X. Tang Y. Hu B. Ou S. (2018). Immunomodulatory Effects of Enzymatic-Synthesized α-Galactooligosaccharides and Evaluation of the Structure-Activity Relationship. *J. Agric. Food Chem.* 66 9070–9079. 10.1021/acs.jafc.8b01939 30086236

[B9] De Almeida C. V. de Camargo M. R. Russo E. Amedei A. (2019). Role of diet and gut microbiota on colorectal cancer immunomodulation. *World J. Gastroenterol.* 25 151–162. 10.3748/wjg.v25.i2.151 30670906 PMC6337022

[B10] Duerr C. U. Hornef M. W. (2012). The mammalian intestinal epithelium as integral player in the establishment and maintenance of host-microbial homeostasis. *Semin. Immunol.* 24 25–35. 10.1016/j.smim.2011.11.002 22138188

[B11] Dwivedi M. Kumar P. Laddha N. C. Kemp E. H. (2016). Induction of regulatory T cells: A role for probiotics and prebiotics to suppress autoimmunity. *Autoimmun. Rev.* 15 379–392. 10.1016/j.autrev.2016.01.002 26774011

[B12] Eiwegger T. Stahl B. Schmitt J. Boehm G. Gerstmayr M. Pichler J. (2004). Human milk–derived oligosaccharides and plant-derived oligosaccharides stimulate cytokine production of cord blood T-cells in vitro. *Pediatr. Res.* 56 536–540. 10.1203/01.pdr.0000139411.35619.b4 15295093

[B13] Farthing M. J. (2004). Bugs and the gut: An unstable marriage. *Best Pract. Res. Clin. Gastroenterol.* 18 233–239. 10.1016/j.bpg.2003.11.001 15123066

[B14] Fedorak R. N. Madsen K. L. (2004). Probiotics and prebiotics in gastrointestinal disorders. *Curr. Opin. Gastroenterol.* 20 146–155. 10.1097/00001574-200403000-00017 15703637

[B15] Fernández J. Moreno F. J. Olano A. Clemente A. Villar C. J. Lombó F. (2018). A Galacto-Oligosaccharides Preparation Derived From Lactulose Protects Against Colorectal Cancer Development in an Animal Model. *Front. Microbiol.* 9:2004. 10.3389/fmicb.2018.02004 30233512 PMC6127505

[B16] Fewtrell M. S. Morgan J. B. Duggan C. Gunnlaugsson G. Hibberd P. L. Lucas A. (2007). Optimal duration of exclusive breastfeeding: What is the evidence to support current recommendations? *Am. J. Clin. Nutr.* 85 635s–638s. 10.1093/ajcn/85.2.635S 17284769

[B17] Figueroa-González I. Quijano G. Ramírez G. Cruz-Guerrero A. (2011). Probiotics and prebiotics–perspectives and challenges. *J. Sci. Food Agric.* 91 1341–1348. 10.1002/jsfa.4367 21445871

[B18] Gasteiger E. Gattiker A. Hoogland C. Ivanyi I. Appel R. D. Bairoch A. (2003). ExPASy: The proteomics server for in-depth protein knowledge and analysis. *Nucleic Acids Res.* 31 3784–3788. 10.1093/nar/gkg563 12824418 PMC168970

[B19] Gibson G. R. Roberfroid M. B. (1995). Dietary modulation of the human colonic microbiota: Introducing the concept of prebiotics. *J. Nutr.* 125 1401–1412. 10.1093/jn/125.6.1401 7782892

[B20] Godínez-Méndez L. A. Gurrola-Díaz C. M. Zepeda-Nuño J. S. Vega-Magaña N. Lopez-Roa R. I. Íñiguez-Gutiérrez L. (2021). In Vivo Healthy Benefits of Galacto-Oligosaccharides from Lupinus albus (LA-GOS) in Butyrate Production through Intestinal Microbiota. *Biomolecules* 11:1658. 10.3390/biom11111658 34827656 PMC8615603

[B21] Gopalakrishnan A. Clinthorne J. F. Rondini E. A. McCaskey S. J. Gurzell E. A. Langohr I. M. (2012). Supplementation with galacto-oligosaccharides increases the percentage of NK cells and reduces colitis severity in Smad3-deficient mice. *J. Nutr.* 142 1336–1342. 10.3945/jn.111.154732 22496400

[B22] Griffin I. J. Davila P. M. Abrams S. A. (2002). Non-digestible oligosaccharides and calcium absorption in girls with adequate calcium intakes. *Br. J. Nutr.* 87:S187–S191. 10.1079/bjnbjn/2002536 12088517

[B23] Hashmi A. Naeem N. Farooq Z. Masood S. Iqbal S. Naseer R. (2016). Effect of Prebiotic Galacto-Oligosaccharides on Serum Lipid Profile of Hypercholesterolemics. *Probiotics Antimicrob. Proteins* 8 19–30. 26905736 10.1007/s12602-016-9206-1

[B24] He N. Chen H. Zhou Z. Zhao W. Wang S. Lv Z. (2021a). Enzymatically synthesized α-galactooligosaccharides attenuate metabolic syndrome in high-fat diet induced mice in association with the modulation of gut microbiota. *Food Funct.* 12 4960–4971. 10.1039/d0fo03113e 34100482

[B25] He N. Wang Y. Zhou Z. Liu N. Jung S. Lee M. S. (2021b). Preventive and Prebiotic Effect of α-Galacto-Oligosaccharide against Dextran Sodium Sulfate-Induced Colitis and Gut Microbiota Dysbiosis in Mice. *J. Agric. Food Chem.* 69 9597–9607. 10.1021/acs.jafc.1c03792 34378931

[B26] Holma R. Juvonen P. Asmawi M. Z. Vapaatalo H. Korpela R. (2002). Galacto-oligosaccharides stimulate the growth of bifidobacteria but fail to attenuate inflammation in experimental colitis in rats. *Scand J. Gastroenterol.* 37 1042–1047. 10.1080/003655202320378239 12374229

[B27] Hong K. B. Jeong M. Han K. S. Hwan Kim J. Park Y. Suh H. J. (2015). Photoprotective effects of galacto-oligosaccharide and/or Bifidobacterium longum supplementation against skin damage induced by ultraviolet irradiation in hairless mice. *Int. J. Food Sci. Nutr.* 66 923–930. 10.3109/09637486.2015.1088823 26470918

[B28] Hsu C. A. Lee S. L. Chou C. C. (2007). Enzymatic production of galactooligosaccharides by beta-galactosidase from Bifidobacterium longum BCRC 15708. *J. Agric. Food Chem.* 55 2225–2230. 10.1021/jf063126 17316019

[B29] Huang S. F. Liu D. B. Zeng J. M. Yuan Y. Xiao Q. Sun C. M. (2009). Cloning, expression, purification, distribution and kinetics characterization of the bacterial beta-galactosidase fused to the cytoplasmic transduction peptide in vitro and in vivo. *Protein Expr. Purif.* 68 167–176. 10.1016/j.pep.2009.06.019 19573604

[B30] Ishikawa H. Matsumoto S. Ohashi Y. Imaoka A. Setoyama H. Umesaki Y. (2011). Beneficial effects of probiotic bifidobacterium and galacto-oligosaccharide in patients with ulcerative colitis: A randomized controlled study. *Digestion* 84 128–133. 10.1159/000322977 21525768

[B31] Jess T. Rungoe C. Peyrin-Biroulet L. (2012). Risk of colorectal cancer in patients with ulcerative colitis: A meta-analysis of population-based cohort studies. *Clin. Gastroenterol. Hepatol.* 10 639–645. 10.1016/j.cgh.2012.01.010 22289873

[B32] Kalliomäki M. Salminen S. Poussa T. Arvilommi H. Isolauri E. (2003). Probiotics and prevention of atopic disease: 4-year follow-up of a randomised placebo-controlled trial. *Lancet* 361 1869–1871. 10.1016/s0140-6736(03)13490-3 12788576

[B33] Kano M. Masuoka N. Kaga C. Sugimoto S. Iizuka R. Manabe K. (2013). Consecutive Intake of Fermented Milk Containing Bifidobacterium breve Strain Yakult and Galacto-oligosaccharides Benefits Skin Condition in Healthy Adult Women. *Biosci. Microbiota Food Health* 32 33–39. 24936360 10.12938/bmfh.32.33 PMC4034291

[B34] Kawakami K. Makino I. Asahara T. Kato I. Onoue M. (2005). Dietary galacto-oligosaccharides mixture can suppress serum phenol and p-cresol levels in rats fed tyrosine diet. *J. Nutr. Sci. Vitaminol.* 51 182–186. 10.3177/jnsv.51.182 16161769

[B35] Kim C. S. Ji E. S. Oh D. K. (2004). Characterization of a thermostable recombinant beta-galactosidase from Thermotoga maritima. *J. Appl. Microbiol.* 97 1006–1014. 10.1111/j.1365-2672.2004.02377.x 15479416

[B36] Kimura L. J. McGee A. Baird S. Viloria J. Nagatsuka M. (2015). Healthy Mothers Healthy Babies: Awareness and perceptions of existing breastfeeding and postpartum depression support among parents and perinatal health care providers in Hawai’i. *Hawaii J. Med. Public Health* 74 101–111. 25821653 PMC4363932

[B37] Knol J. Scholtens P. Kafka C. Steenbakkers J. Gro S. Helm K. (2005). Colon microflora in infants fed formula with galacto- and fructo-oligosaccharides: More like breast-fed infants. *J. Pediatr. Gastroenterol. Nutr.* 40 36–42. 10.1097/00005176-200501000-00007 15625424

[B38] Kukkonen K. Savilahti E. Haahtela T. Juntunen-Backman K. Korpela R. Poussa T. (2007). Probiotics and prebiotic galacto-oligosaccharides in the prevention of allergic diseases: A randomized, double-blind, placebo-controlled trial. *J. Allergy Clin. Immunol.* 119 192–198. 10.1016/j.jaci.2006.09.009 17208601

[B39] Li Y. Lu L. Wang H. Xu X. Xiao M. (2009). Cell surface engineering of a beta-galactosidase for galactooligosaccharide synthesis. *Appl. Environ. Microbiol.* 75 5938–5942. 10.1128/aem.00326-09 19617384 PMC2747861

[B40] Lu L. Guo L. Wang K. Liu Y. Xiao M. (2020). β-Galactosidases: A great tool for synthesizing galactose-containing carbohydrates. *Biotechnol. Adv.* 39:107465. 10.1016/j.biotechadv.2019.107465 31689470

[B41] Macfarlane G. T. Steed H. Macfarlane S. (2008). Bacterial metabolism and health-related effects of galacto-oligosaccharides and other prebiotics. *J. Appl. Microbiol.* 104 305–344. 10.1111/j.1365-2672.2007.03520.x 18215222

[B42] Marín-Manzano M. C. Abecia L. Hernández-Hernández O. Sanz M. L. Montilla A. Olano A. (2013). Galacto-oligosaccharides derived from lactulose exert a selective stimulation on the growth of Bifidobacterium animalis in the large intestine of growing rats. *J. Agric. Food Chem.* 61 7560–7567. 10.1021/jf402218z 23855738

[B43] Mistry R. H. Liu F. Borewicz K. Lohuis M. A. M. Smidt H. Verkade H. J. (2020). Long-Term β-galacto-oligosaccharides Supplementation Decreases the Development of Obesity and Insulin Resistance in Mice Fed a Western-Type Diet. *Mol. Nutr. Food Res.* 64:e1900922. 10.1002/mnfr.201900922 32380577 PMC7379190

[B44] Moro G. Arslanoglu S. Stahl B. Jelinek J. Wahn U. Boehm G. (2006). A mixture of prebiotic oligosaccharides reduces the incidence of atopic dermatitis during the first six months of age. *Arch Dis. Child.* 91 814–819. 10.1136/adc.2006.098251 16873437 PMC2066015

[B45] Moro G. Minoli I. Mosca M. Fanaro S. Jelinek J. Stahl B. (2002). Dosage-related bifidogenic effects of galacto- and fructooligosaccharides in formula-fed term infants. *J. Pediatr. Gastroenterol. Nutr.* 34 291–295. 10.1097/00005176-200203000-00014 11964956

[B46] Ordás I. Eckmann L. Talamini M. Baumgart D. C. Sandborn W. J. (2012). Ulcerative colitis. *Lancet* 380 1606–1619. 10.1016/s0140-6736(12)60150-0 22914296

[B47] Otieno D. O. (2010). Synthesis of β-Galactooligosaccharides from Lactose Using Microbial β-Galactosidases. *Compr. Rev. Food Sci. Food Saf.* 9 471–482. 10.1111/j.1541-4337.2010.00121.x 33467831

[B48] Panesar P. S. Kaur R. Singh R. S. Kennedy J. F. (2018). Biocatalytic strategies in the production of galacto-oligosaccharides and its global status. *Int. J. Biol. Macromol.* 111 667–679. 10.1016/j.ijbiomac.2018.01.062 29339290

[B49] Park A. R. Oh D. K. (2010a). Effects of galactose and glucose on the hydrolysis reaction of a thermostable beta-galactosidase from Caldicellulosiruptor saccharolyticus. *Appl. Microbiol. Biotechnol.* 85 1427–1435. 10.1007/s00253-009-2165-7 19662397

[B50] Park A. R. Oh D. K. (2010b). Galacto-oligosaccharide production using microbial beta-galactosidase: Current state and perspectives. *Appl. Microbiol. Biotechnol.* 85 1279–1286. 10.1007/s00253-009-2356-2 19943044

[B51] Park H. R. Eom D. H. Kim J. H. Shin J. C. Shin M. S. Shin K. S. (2021). Composition analysis and oral administered effects on dextran sulfate sodium-induced colitis of galactooligosaccharides bioconverted by Bacillus circulans. *Carbohydr. Polym.* 270:118389. 10.1016/j.carbpol.2021.118389 34364630

[B52] Peguet-Navarro J. Dezutter-Dambuyant C. Buetler T. Leclaire J. Smola H. Blum S. (2008). Supplementation with oral probiotic bacteria protects human cutaneous immune homeostasis after UV exposure-double blind, randomized, placebo controlled clinical trial. *Eur. J. Dermatol.* 18 504–511. 10.1684/ejd.2008.0496 18693151

[B53] Pignatelli R. Vai M. Alberghina L. Popolo L. (1998). Expression and secretion of beta-galactosidase in Saccharomyces cerevisiae using the signal sequences of GgpI, the major yeast glycosylphosphatidylinositol-containing protein. *Biotechnol. Appl. Biochem.* 27 81–88. 10.1111/j.1470-8744.1998.tb01378.x 9569602

[B54] Roselli M. Maruszak A. Grimaldi R. Harthoorn L. Finamore A. (2022). Galactooligosaccharide Treatment Alleviates DSS-Induced Colonic Inflammation in Caco-2 Cell Model. *Front. Nutr.* 14:862974. 10.3389/fnut.2022.862974 35495925 PMC9047546

[B55] Rossi M. Mirbagheri S. Keshavarzian A. Bishehsari F. (2018). Nutraceuticals in colorectal cancer: A mechanistic approach. *Eur. J. Pharmacol.* 833 396–402. 10.1016/j.ejphar.2018.06.027 29935172 PMC6063737

[B56] Sangwan V. Tomar S. K. Singh R. R. Singh A. K. Ali B. (2011). Galactooligosaccharides: Novel components of designer foods. *J. Food Sci.* 76:R103–R111. 10.1111/j.1750-3841.2011.02131.x 22417365

[B57] Schmelzle H. Wirth S. Skopnik H. Radke M. Knol J. Böckler H. M. (2003). Randomized double-blind study of the nutritional efficacy and bifidogenicity of a new infant formula containing partially hydrolyzed protein, a high beta-palmitic acid level, and nondigestible oligosaccharides. *J. Pediatr. Gastroenterol. Nutr.* 36 343–351. 10.1097/00005176-200303000-00008 12604972

[B58] Scholz-Ahrens K. E. Açil Y. Schrezenmeir J. (2002). Effect of oligofructose or dietary calcium on repeated calcium and phosphorus balances, bone mineralization and trabecular structure in ovariectomized rats. *Br. J. Nutr.* 88 365–377. 10.1079/bjn2002661 12323086

[B59] Sheveleva S. A. (1999). [Probiotics, prebiotics and probiotic products. Current status]. *Vopr. Pitan.* 68 32–40. 10224650

[B60] Shoaf K. Mulvey G. L. Armstrong G. D. Hutkins R. W. (2006). Prebiotic galactooligosaccharides reduce adherence of enteropathogenic *Escherichia coli* to tissue culture cells. *Infect. Immun.* 74 6920–6928. 10.1128/iai.01030-06 16982832 PMC1698067

[B61] Sierra C. Bernal M. J. Blasco J. Martínez R. Dalmau J. Ortuño I. (2015). Prebiotic effect during the first year of life in healthy infants fed formula containing GOS as the only prebiotic: A multicentre, randomised, double-blind and placebo-controlled trial. *Eur. J. Nutr.* 54 89–99. 24671237 10.1007/s00394-014-0689-9 PMC4303717

[B62] Sun J. Liang W. Yang X. Li Q. Zhang G. (2019). Cytoprotective effects of galacto-oligosaccharides on colon epithelial cells via up-regulating miR-19b. *Life Sci.* 231:116589. 10.1016/j.lfs.2019.116589 31226416

[B63] Tanabe S. Hochi S. (2010). Oral administration of a galactooligosaccharide preparation inhibits development of atopic dermatitis-like skin lesions in NC/Nga mice. *Int. J. Mol. Med.* 25 331–336. 10.3892/ijmm_00000349 20127036

[B64] Teuri U. Korpela R. (1998). Galacto-oligosaccharides relieve constipation in elderly people. *Ann. Nutr. Metab.* 42 319–327. 10.1159/000012751 9895419

[B65] Tian S. Wang J. Yu H. Wang J. Zhu W. (2019). Changes in Ileal Microbial Composition and Microbial Metabolism by an Early-Life Galacto-Oligosaccharides Intervention in a Neonatal Porcine Model. *Nutrients* 11:1753. 10.3390/nu11081753 31366090 PMC6723927

[B66] Torres D. P. M. Gonçalves M. Teixeira J. A. Rodrigues L. R. (2010). Galacto-Oligosaccharides: Production, Properties, Applications, and Significance as Prebiotics. *Compr. Rev. Food Sci. Food Saf.* 9 438–454. 10.1111/j.1541-4337.2010.00119.x 33467830

[B67] van Hoffen E. Ruiter B. Faber J. M’Rabet L. Knol E. F. Stahl B. (2009). A specific mixture of short-chain galacto-oligosaccharides and long-chain fructo-oligosaccharides induces a beneficial immunoglobulin profile in infants at high risk for allergy. *Allergy* 64 484–487. 10.1111/j.1398-9995.2008.01765.x 18507650

[B68] Varney J. Barrett J. Scarlata K. Catsos P. Gibson P. R. Muir J. G. (2017). FODMAPs: Food composition, defining cutoff values and international application. *J. Gastroenterol. Hepatol.* 32 53–61. 10.1111/jgh.13698 28244665

[B69] Vera C. Córdova A. Aburto C. Guerrero C. Suárez S. Illanes A. (2016). Synthesis and purification of galacto-oligosaccharides: State of the art. *World J. Microbiol. Biotechnol.* 32:197. 10.1007/s11274-016-2159-4 27757792

[B70] Vulevic J. Drakoularakou A. Yaqoob P. Tzortzis G. Gibson G. R. (2008). Modulation of the fecal microflora profile and immune function by a novel trans-galactooligosaccharide mixture (B-GOS) in healthy elderly volunteers. *Am. J. Clin. Nutr.* 88 1438–1446. 10.3945/ajcn.2008.26242 18996881

[B71] Vulevic J. Juric A. Walton G. E. Claus S. P. Tzortzis G. Toward R. E. (2015). Influence of galacto-oligosaccharide mixture (B-GOS) on gut microbiota, immune parameters and metabonomics in elderly persons. *Br. J. Nutr.* 114 586–595. 10.1017/s0007114515001889 26218845

[B72] Walton G. E. van den Heuvel E. G. Kosters M. H. Rastall R. A. Tuohy K. M. Gibson G. R. (2012). A randomised crossover study investigating the effects of galacto-oligosaccharides on the faecal microbiota in men and women over 50 years of age. *Br. J. Nutr.* 107 1466–1475. 10.1017/s0007114511004697 21910949

[B73] Weaver C. M. Martin B. R. Nakatsu C. H. Armstrong A. P. Clavijo A. McCabe L. D. (2011). Galactooligosaccharides improve mineral absorption and bone properties in growing rats through gut fermentation. *J. Agric. Food Chem.* 59 6501–6510. 10.1021/jf2009777 21553845

[B74] Weijers C. A. Franssen M. C. Visser G. M. (2008). Glycosyltransferase-catalyzed synthesis of bioactive oligosaccharides. *Biotechnol. Adv.* 26 436–456. 10.1016/j.biotechadv.2008.05.001 18565714

[B75] Wilson B. Eyice Ö Koumoutsos I. Lomer M. C. Irving P. M. Lindsay J. O. (2021). Prebiotic Galactooligosaccharide Supplementation in Adults with Ulcerative Colitis: Exploring the Impact on Peripheral Blood Gene Expression, Gut Microbiota, and Clinical Symptoms. *Nutrients* 13:3598. 10.3390/nu13103598 34684597 PMC8537576

[B76] Wilson B. Whelan K. (2017). Prebiotic inulin-type fructans and galacto-oligosaccharides: Definition, specificity, function, and application in gastrointestinal disorders. *J. Gastroenterol. Hepatol.* 32 64–68. 10.1111/jgh.13700 28244671

[B77] Xiao Y. Chen Q. Guang C. Zhang W. Mu W. (2019). An overview on biological production of functional lactose derivatives. *Appl. Microbiol. Biotechnol.* 103 3683–3691. 10.1007/s00253-019-09755-6 30911789

[B78] Yang S. T. Silva E. M. (1995). Novel products and new technologies for use of a familiar carbohydrate, milk lactose. *J. Dairy Sci.* 78 2541–2562. 10.3168/jds.S0022-0302(95)76884-9 8747342

[B79] Zhong Y. Cai D. Cai W. Geng S. Chen L. Han T. (2009). Protective effect of galactooligosaccharide-supplemented enteral nutrition on intestinal barrier function in rats with severe acute pancreatitis. *Clin. Nutr.* 28 575–580. 10.1016/j.clnu.2009.04.026 19525042

